# Elevated serum levels of HIF-1α and VEGF as potential biomarkers in connective tissue disease-associated pulmonary arterial hypertension

**DOI:** 10.1038/s41598-025-89130-w

**Published:** 2025-02-13

**Authors:** Xiaofei Shi, Xin Ma, Huanhuan Wang, Xuegai He, Hua Fan, Yimin Mao

**Affiliations:** 1 Department of Rheumatology and Immunology, The First Affiliated Hospital, and College of Clinical Medicine of Henan University of Science and Technology, Luoyang, China; 2 Department of Respiratory and Critical Care Medicine, People’s Hospital of Zhengzhou, Zhengzhou, China; 3 Department of Respiratory and Critical Care Medicine, The First Affiliated Hospital, and College of Clinical Medicine of Henan University of Science and Technology, Luoyang, China; 4 Office of Research & Innovation, The First Affiliated Hospital, and College of Clinical Medicine of Henan University of Science and Technology, Luoyang, China

**Keywords:** Connective tissue disease, Pulmonary arterial hypertension, Hypoxia-inducible Factor-1α, Vascular endothelial growth factor, 6-Minute walk distance, Immunology, Biomarkers, Cardiology, Rheumatology

## Abstract

We to measure the serum levels of hypoxia-inducible factor-1α (HIF-1α) and vascular endothelial growth factor (VEGF) in patients diagnosed with connective tissue disease (CTD)-associated pulmonary arterial hypertension (PAH) (CTD-PAH) and to analyze their clinical implications. Thirty patients who were diagnosed with CTD-PAH via right heart catheterization (RHC) were enrolled in the observation group. Additionally, twenty CTD patients without PAH (CTD-non-PAH) were enrolled in the CTD-non-PAH group, and twenty healthy participants were enrolled in the healthy control group. Additional data on CTD-PAH patients, including demographic characteristics, laboratory parameters, and hemodynamic measurements, were obtained. The serum levels of HIF-1α and VEGF in all participants were measured using an enzyme-linked immunosorbent assay (ELISA). Pearson correlation was utilized to determine the correlation between the expression levels of these factors and clinical data. Receiver operating characteristic (ROC) curves were constructed, and the area under the curve (AUC) was calculated to evaluate the diagnostic efficacy of expression of HIF-1α and VEGF for diagnosing CTD-PAH. Participants in the three groups were age-matched. The CTD-PAH group included 29 females and 1 male, and systemic lupus erythematosus (SLE) was the most prevalent CTD diagnosis (17/30). Serum levels of VEGF and HIF-1α were significantly greater in both CTD-PAH and CTD-non-PAH patients compared to the healthy controls (*P* < 0.01). In addition, the serum concentration of HIF-1α in the CTD-PAH group was significantly greater than that in the CTD-non-PAH group (248.86 ± 18.85 and 224.64 ± 12.22, respectively, *P* = 0.0022). Similarly, the serum concentration of VEGF in the CTD-PAH group was significantly greater than that in the CTD-non-PAH group (251.11 ± 48.37 and 222.120 ± 18.57, respectively, *P* = 0.0026). In patients with CTD-PAH, the concentrations of HIF-1α and VEGF are significantly positively correlated with mPAP (*r* = 0.81, *r* = 0.88). Both HIF-1α and VEGF concentrations were positively correlated with BNP levels (*r* = 0.5340, *r* = 0.6472) and negatively correlated with the 6-Minute Walk Distance (6MWD) (*r*=-0.46, *r*=-0.47, respectively, *P* < 0.01). When VEGF and HIF-1α were combined, the diagnostic ROC curve yielded an AUC of 0.933. Increased serum levels of HIF-1α and VEGF were observed in CTD-PAH patients compared to CTD-non-PAH patients and healthy controls, which, along with their positive correlations with mPAP and BNP levels and negative correlations with the 6MWD, indicate their potential as biomarkers for diagnosing CTD-PAH.

**Supplementary Information:**

The online version contains supplementary material available at 10.1038/s41598-025-89130-w.

## Introduction

Pulmonary arterial hypertension (PAH) poses a grave risk of heart failure or mortality. Diagnosis typically requires right heart catheterization (RHC) to measure a mean pulmonary arterial pressure (mPAP) exceeding 20 mmHg at rest^1^. PAH is a common complication of connective tissue disease (CTD) and significantly increases mortality risk^2^. Although various diagnostic modalities, such as electrocardiography, chest CT, pulmonary function tests, echocardiography, and cardiac MRI, aid in PAH diagnosis, RHC remains the gold standard. Early diagnosis remains elusive, with studies indicating that approximately half of CTD-associated PAH (CTD-PAH) patients present at WHO functional class III to IV, with some already experiencing irreversible cardiac damage^3–7^. Noninvasive plasma biomarkers could aid in early diagnosis and improve outcomes. Although B-type natriuretic peptide (BNP) and N-terminal pro-b-type natriuretic peptide (NT-proBNP) are related to PAH and aid in diagnosis and prognosis, they do not indicate the disease’s etiology[8]. Studies suggest that plasma protein biomarkers associated with vascular abnormalities and cardiac injury, such as hypoxia-inducible factor-1α (HIF-1α) and vascular endothelial growth factor (VEGF), are prominently expressed in the endothelial cells of plexiform lesions in severe PAH^9^.

CTD-PAH involves immune-mediated pulmonary vasculitis that leads to pulmonary vascular changes and vascular remodeling under hypoxic conditions. Vasculitis induces pulmonary vascular structure changes and lung vasoconstriction and alters immune cell and cytokine functions and actions under hypoxic conditions^10^. Hypoxia is a crucial instigator of pathological changes in CTD-PAH^11^. In CTD-PAH, the interplay between HIF-1α and VEGF likely fosters pathological proliferation and migration of vascular smooth muscle and endothelial cells, culminating in pulmonary vascular remodeling and heightened pulmonary vascular resistance (PVR). In 1992, Semenza et al. characterized HIF-1 as a heterodimeric transcription factor comprising α and β subunits found within cell nuclei under hypoxic conditions^12^. HIF-1α subunit expression is modulated by oxygen levels and is crucial for regulating HIF-1 activity under hypoxic conditions, whereas HIF-1β is ubiquitously expressed^12^. HIF-1 plays significant roles in various pulmonary conditions including the pathogenesis of CTD-PAH^13^. The VEGF family and its receptors are pivotal in pulmonary vascular remodeling^14^. Under hypoxic conditions, the protein kinase C signaling pathway induces VEGF production, promoting smooth muscle cell proliferation via increased endothelin-1 production in vascular smooth muscle cells, thus triggering pulmonary vascular remodeling and elevated PAP^15^. Studies indicate that HIF-1α can enhance the hypoxic response of endothelial cells by upregulating VEGF expression, contributing to vasoconstriction and pulmonary vessel remodeling in CTD-PAH patients.

Accumulating evidence underscores the potential significance of HIF-1α and VEGF as key pathogenic elements in CTD-PAH, potentially influencing disease development. Nonetheless, clinical studies examining the correlation among VEGF, HIF-1α, and pulmonary arterial pressure (PAP) in CTD-PAH patients are scarce. Therefore, it is critical to explore the expression levels of HIF-1α and VEGF in CTD-PAH patients and their associations with hemodynamic parameters. This study investigated the serum levels of HIF-1α and VEGF in CTD-PAH patients and analyzed their clinical relevance, aiming to establish HIF-1α and VEGF as potential diagnostic biomarkers for CTD-PAH.

## Materials and methods

### Study subjects

This single-center retrospective cohort study enrolled 124 patients who were diagnosed with CTD-PAH at the Department of Rheumatology and Immunology at the First Affiliated Hospital of Henan University of Science and Technology between October 1, 2020, and October 31, 2023. Patients who lacked echocardiography at our institution were excluded, resulting in the exclusion of 52 individuals. Among the remaining cohort, 72 patients with CTD-PAH were identified via echocardiography. Of these, 45 patients had an mPAP ≥ 20 mmHg, as measured by RHC, meeting the diagnostic criteria for PAH according to the 2022 ESC/ERS guidelines on pulmonary hypertension. These patients were diagnosed with CTD based on the American College of Rheumatology or corresponding international diagnostic criteria. After excluding 15 patients due to missing or incomplete data, a total of 30 patients with CTD-PAH were included in the study. As controls, 20 CTD patients without PAH (CTD-non-PAH), matched by age and sex, were included. These patients had pulmonary arterial systolic pressure ≤ 36 mmHg, assessed via echocardiography, and no other clinical manifestations of PAH. Additionally, 20 healthy volunteers (without CTD or PAH) were selected as the healthy control group (HC). Neither control group underwent invasive RHC.

### Inclusion criteria

1. Patients who underwent RHC, where PAH was defined according to the 2022 ESC/ERS PH guidelines as an mPAP > 20 mmHg, at sea level at rest, with a pulmonary arterial wedge pressure (PAWP) ≤ 15 mmHg and PVR > 2 Wood units (WUs)^1^.
2. All patients diagnosed with CTD met the corresponding disease classification criteria, including systemic lupus erythematosus (SLE)^16^, Sjögren’s syndrome (SS)^17^, rheumatoid arthritis (RA)^18^, systemic sclerosis (SSc)^19^, and mixed CTD (MCTD)^20^.
3. Patients ≥ 18 years old.
4. Patients had not received any oral PAP-lowering targeted medicine before blood samples were collected.

### Exclusion criteria

1. Patients diagnosed with postcapillary pulmonary hypertension according to the RHC results, diagnosed with other types of precapillary pulmonary hypertension.
2. Patients diagnosed with HIV infection or portal hypertension.
3. Patients with a history of or concurrent other active malignancies.

## Methods

### Data

The demographic and clinical data, RHC hemodynamics, serological characteristics, and serum concentrations of HIF-1α and VEGF in patients with CTD-PAH were recorded in detail, including (1) clinical data including name, gender, age, height, weight, type of CTD, duration of CTD, 6-Minute Walk Distance (6MWD), BNP levels, ANA profile, date of visit, medication usage, and involvement of other organs; (2) RHC hemodynamics data, including mPAP, right atrial pressure (RAP), cardiac index (CI), and PVR; (3) serum concentrations of HIF-1α and VEGF in venous blood serum detected by enzyme-linked immunosorbent assay (ELISA). Serum samples from all enrolled patients were obtained within one month, and approximately 5 mL of peripheral venous blood was collected. The samples were centrifuged at 3000 r/min for 25 min using a GeneSpeed micro high-speed refrigerated centrifuge at room temperature, and the supernatant was stored at -80 °C in a freezer (Thermo Fisher Scientific, USA) for future use. Expression levels were determined using a human HIF-1α ELISA kit from Thermo Fisher Scientific (USA) and a human VEGF-A ELISA kit from RayBiotech (USA).

### Statistical analysis

Statistical analysis was performed using GraphPad Prism 10.0 software. Descriptive statistics for continuous variables were presented as mean and standard deviation (SD) or as median and interquartile range (IQR), depending on the data distribution. Categorical variables were described using numbers and percentages. Differences among multiple groups were analyzed using one-way ANOVA, and differences between two groups were analyzed using the t test. Pearson correlation analysis was used when both variables in the comparison groups were normally distributed; Spearman correlation analysis was used when either variable in any group was not normally distributed or when both variables in the comparison groups were not normally distributed. Receiver operating characteristic (ROC) curves were constructed, and the area under the curve (AUC) was calculated to evaluate the diagnostic efficacy of expression of HIF-1α and VEGF for diagnosing CTD-PAH. A *P* value < 0.05 was considered statistically significant.

## Research results

### Clinical characteristics of the CTD-PAH Group

The characteristics of the 30 patients in the CTD-PAH group are described in Table 1. Twenty-nine patients were female, and one was male, with an average age of 45.87 ± 14.16 years. The classification of CTD diseases showed that SLE was the most common, accounting for 56.7%, followed by MCTD at 16.7%. RA and SS were equally represented, each accounting for 10%, whereas SSc comprised the remaining 6.7%. All CTD-PAH patients had an mPAP > 20 mmHg, PAWP ≤ 15 mmHg, and PVR > 2 WUs as measured by RHC, meeting the diagnostic criteria for CTD-PAH. Common clinical manifestations included Raynaud’s phenomenon (46.6%), renal involvement (40.0%), and arthritis (40.0%). The ANA profile showed a U1RNP-positive ratio of 46.7%.

**Table 1:** Clinical characteristics of patients in the CTD-PAH Group.

Clinical Characteristics	CTD-PAH(*n* = 30)	CTD-non-PAH(20)	*P*	HCs(*n* = 20)
Age (years)	45.87 ± 14.16	45.36 ± 13.44	0.831	41.89 ± 18.81
Gender (Male/Female)	1/29	0.052631579		0/20
BMI	21.75 ± 3.44	23.67 ± 5.61		20.48 ± 4.65
Blood Pressure (mmHg)				
Systolic	112.5 ± 16.10	120.5 ± 18.54	0.440	118.8 ± 12.72
Diastolic	75.71 ± 10.95	75.71 ± 11.35	0.504	69.98 ± 11.87
Six-Minute Walk Distance (m)	369.4 ± 100.97	398.7 ± 113.34	0.416	610.3 ± 98.32
Right Heart Catheter Data				
mPAP (mmHg)	36.07 ± 11.09	/	/	/
RAP (mmHg)	7.5(3–10)	/	/	/
PVR (WU)	6.44(4.14–9.74)	/	/	/
CI (L/min/m2)	2.53 ± 0.63	/	/	/
CTD Disease Classification (%)			0.965	
SLE	17 (56.67%)	12(60%)		/
SSc	2 (6.67%)	2(10%)		/
RA	3 (10%)	1(5%)		/
MCTD	5 (16.67%)	3(15%)		/
pSS	3 (10%)	2(10%)		/
Clinical Symptoms (%)			0.983	
Raynaud’s Phenomenon	14 (46.6%)	8(40%)		/
Renal Involvement	12 (40.0%	7(35%)		/
Rash	5 (16.7%)	4(20%)		/
Pericardial Effusion	8 (26.7%)	6(30%)		/
Arthritis	12 (40.0%)	9(45%)		/

Note: ①BNP: B-type Natriuretic Peptide; ②mPAP: Mean Pulmonary Arterial Pressure; ③RAP: Right Atrial Pressure; ④CI: Cardiac Index; ⑤PVR: Pulmonary Vascular Resistance; ⑥CTD: Connective Tissue Disease; ⑦SLE: Systemic Lupus Erythematosus; ⑧SSc: Systemic Sclerosis; ⑨RA: Rheumatoid Arthritis; ⑩MCTD: Mixed Connective Tissue Disease; ⑪SS: Sjögren’s Syndrome;⑫6MWD: 6-Minute Walk Distance.

### Serum levels of HIF-1α and VEGF in the three groups

**Fig. 1:**
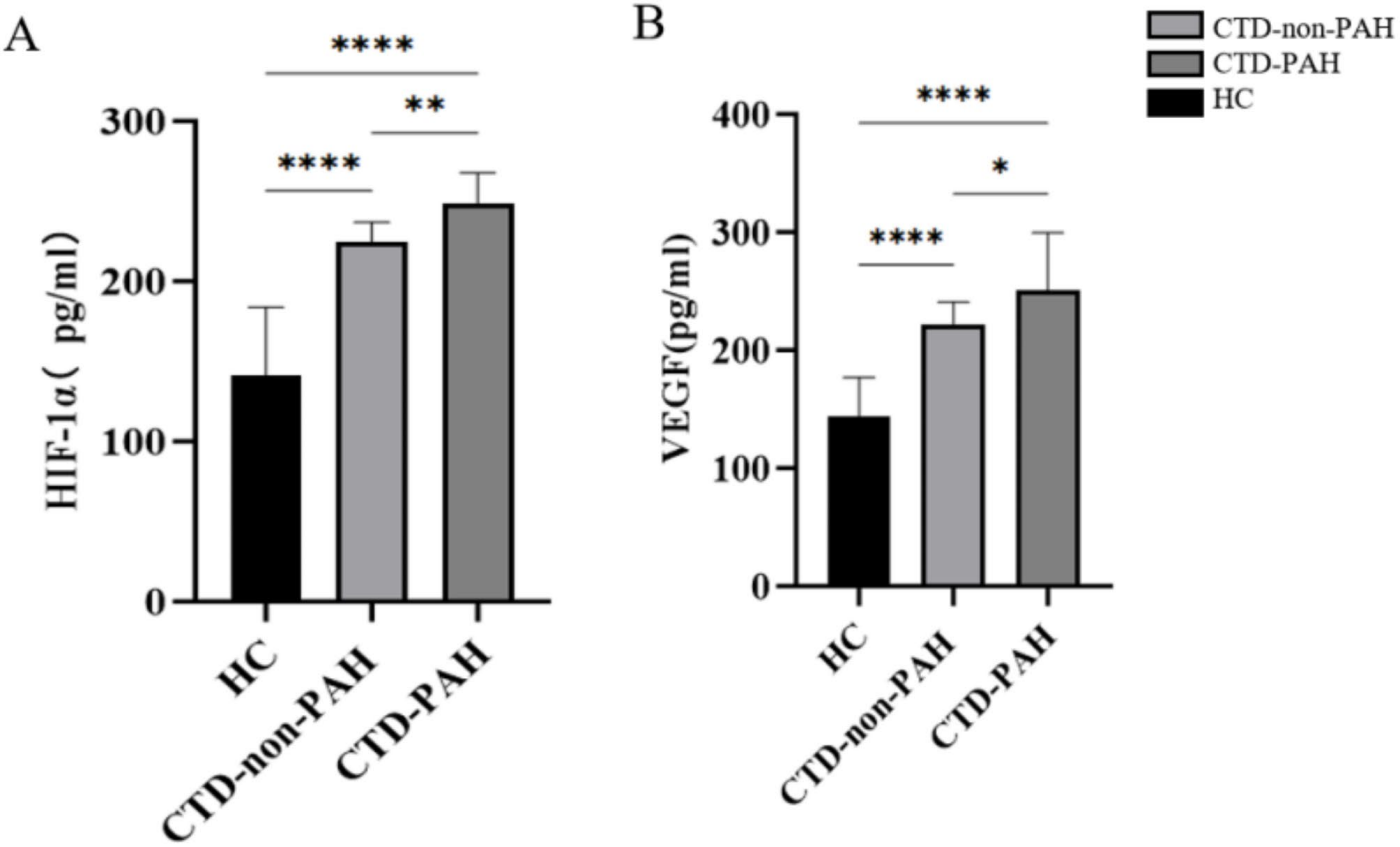
Serum HIF-1α and VEGF concentrations in the three groups of patients.

Table 2 displays the serum levels of HIF-1α and VEGF in the three groups. Statistical analysis revealed that the concentration of HIF-1α in the serum of CTD-PAH patients was significantly greater than that in either the HCs (248.9 ± 18.9 vs. 141 ± 42.9, *P* < 0.01) or the CTD-non-PAH patients (248.9 ± 18.9 vs. 224.6 ± 12.2, *P* < 0.01) (Fig. 1A). The serum VEGF concentration in the CTD-PAH group was significantly greater than that in the HC group (251.1 ± 48.4 vs. 143.5 ± 33.8, *P* < 0.0001) and the CTD-non-PAH group (251.1 ± 48.4 vs. 222.1 ± 18.6, *P* = 0.0026) (Fig. 1B).

**Table 2:** Serum HIF-1α and VEGF concentrations across two patient groups.

Group	HIF-1α (pg/mL)	VEGF (pg/mL)
HC Group(*n* = 20)	141.0 ± 42.9	143.5 ± 33.8
CTD-non-PAH Group (*n* = 20)	224.6 ± 12.2	222.1 ± 18.6
CTD-PAH Group(*n* = 30)	248.9 ± 18.9	251.1 ± 48.4
*F* value	49.4	100.9
*P* value	<0.0001	<0.0001

### Correlation analysis between serum HIF-1α concentration and mPAP, mRAP (mean right atrial pressure), PVR, and CI in CTD-PAH patients

**Fig. 2:**
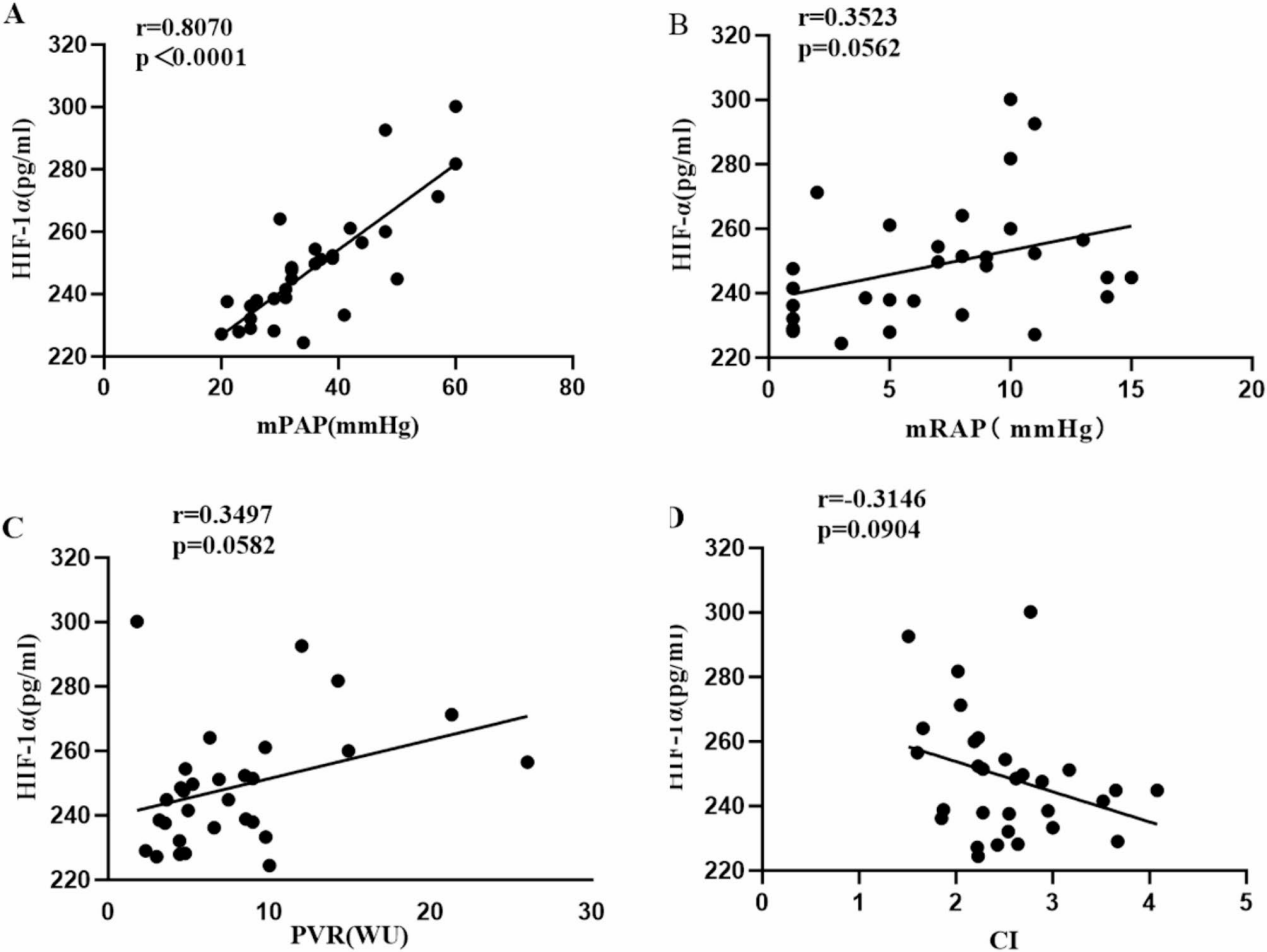
Correlation analysis of the correlation between serum HIF-1α concentration and mPAP, mRAP, PVR, and CI in patients with CTD-PAH.

Spearman correlation analysis revealed that the serum concentration of HIF-1α in CTD-PAH patients was significantly positively correlated with the mPAP (*r* = 0.8070, *P* < 0.0001) (Fig. 2A). It also showed a positive correlation with mRAP (*r* = 0.3523, *P* = 0.0562) and PVR (*r* = 0.3497, *P* = 0.0582) (Fig. 2B and C). However, no correlation was observed with CI (*r*=-0.3146, *P* = 0.0904) (Fig. 2D).

###  Correlation analysis between serum VEGF concentration and mPAP, mRAP, PVR, and CI in CTD-PAH patients

**Fig. 3:**
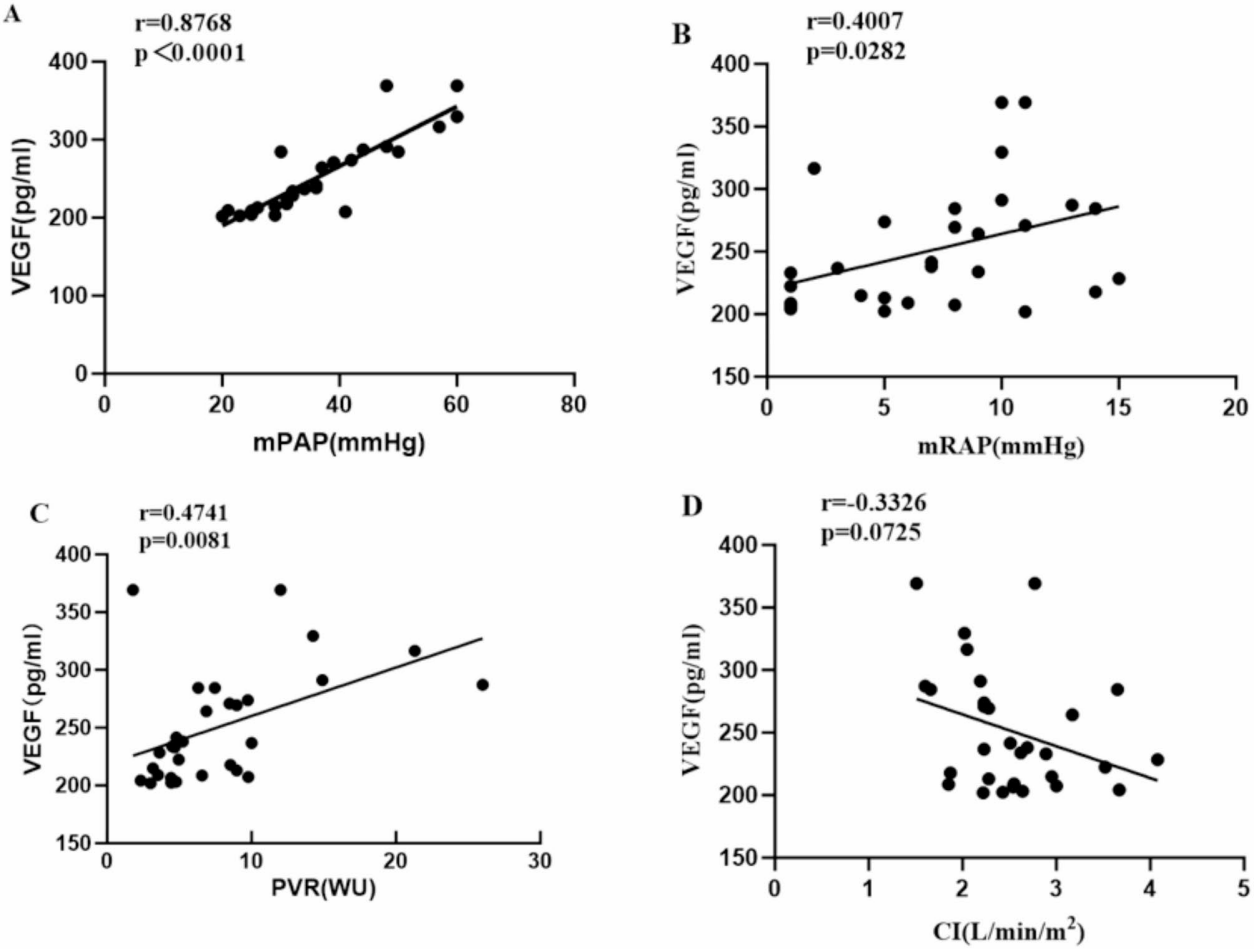
Correlation analysis of the correlation between serum VEGF concentration and mPAP, mRAP, PVR, and CI in patients with CTD-PAH.

Spearman correlation analysis revealed that the serum VEGF concentration in CTD-PAH patients was significantly positively correlated with the mPAP (*r* = 0.8768, *P* < 0.0001) (Fig. 3A). It was also positively correlated with mRAP (*r* = 0.4007, *P* = 0.0282) (Fig. 3B) and PVR (*r* = 0.4741, *P* = 0.0081) (Fig. 3C). Similar to HIF-1α, no correlation was observed with CI (Fig. 3D).

###  Correlation analysis between serum HIF-1α and VEGF concentrations and BNP and the 6MWD in CTD-PAH patients

**Fig. 4:**
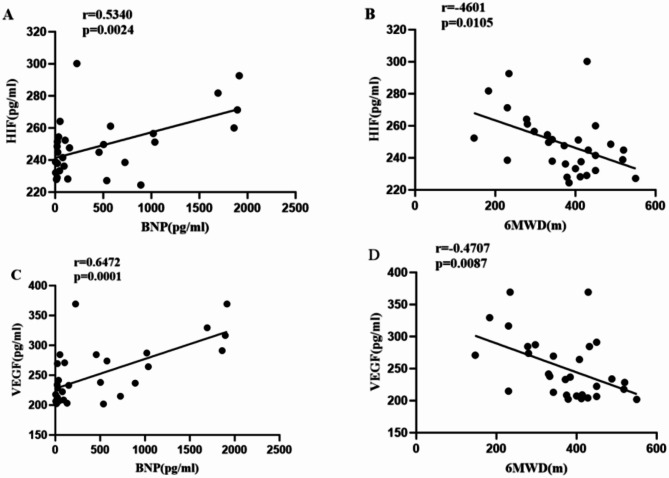
Correlation analysis between serum HIF-1α and VEGF concentrations and BNP and the 6MWD in CTD-PAH patients.

Spearman correlation analysis revealed that in CTD-PAH patients, both serum HIF-1α and VEGF concentrations were significantly positively correlated with BNP levels (*r* = 0.5340 and *r* = 0.6472, respectively) (Fig. 4A and C). These parameters were negatively correlated with the 6MWD (*r*=-0.4601 and *r*=-0.4707, respectively) (Fig. 4B and D), and both correlations were statistically significant.

###  Diagnostic efficacy of HIF-1α and VEGF concentrations, and the combined evaluation in CTD-PAH patients

**Fig. 5:**
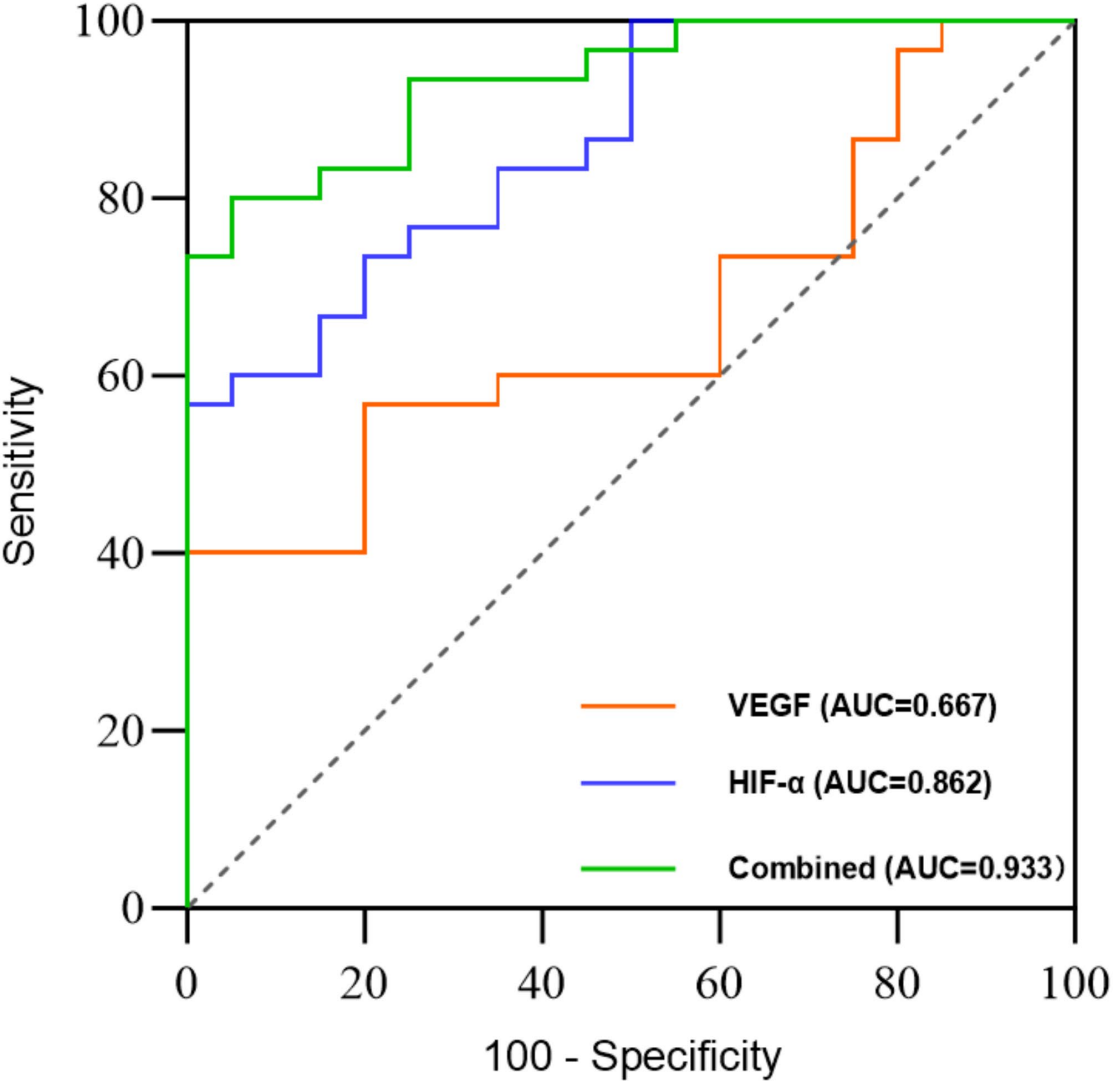
Diagnostic efficacy of HIF-1α and VEGF concentrations, and the combined evaluation in CTD-PAH patients.

The results indicate that there are significant differences in the expression of HIF-1α and VEGF in the serum of patients with CTD-PAH, and these differences are correlated with the mPAP. The diagnostic value of HIF-1α and VEGF in diagnosing CTD-PAH was assessed using ROC curves. The AUC for VEGF in the diagnosis of CTD-PAH was 0.667, whereas the AUC for HIF-1α was 0.862. The optimal threshold The optimal concentration for VEGF was VRGF: 227.21pg/mL, with a sensitivity of 60% and a specificity of 65%; The optimal concentration for HIF-1α was 236,35pg/mL, with a sensitivity of 73% and a specificity of 75%. When VEGF and HIF-1α were combined, the diagnostic ROC curve yielded an AUC of 0.933, suggesting that the combined diagnosis may provide a better predictive value (Fig. 5). In addition, the diagnostic performance of BNP was analyzed using ROC curves, and the results showed that HIF-1 α and VEGF combined exhibited a more significant diagnostic ability and advantages (Supplementary Fig). The AUC for BNP was 0.67.

### Discussion and conclusion

CTD-PAH predominantly affects young women, especially those with SLE, often accompanied by Raynaud’s phenomenon. In our study, the expression levels of HIF-1α and VEGF were significantly elevated in CTD-PAH patients compared to those in the other groups and were positively correlated with the mPAP. Additionally, both biomarkers exhibited positive correlations with BNP levels and negative correlations with the 6MWD, indicating their potential as diagnostic biomarkers for predicting PAP in CTD-PAH patients. CTD-PAH encompasses a spectrum of CTDs and is associated with various clinical manifestations, including SSc, SLE, SS, and MCTD. Although SSc-associated PAH is most common globally^15^, SLE-PAH is more common in China^21^. Our study cohort was predominantly comprised of young female patients with SLE exhibiting typical clinical features such as Raynaud’s phenomenon, renal involvement, arthritis, and pericardial effusion. The majority of patients fell into the low- to intermediate-risk category, consistent with previous reports.

Early diagnosis of CTD-PAH remains challenging due to its insidious onset and complex pathogenesis, leading to delayed treatment and poor outcomes. Hypoxia-induced pulmonary vascular remodeling is a key pathological mechanism in CTD-PAH, and HIF-1α plays a pivotal role in this process. Our findings align with studies in neonatal hypoxic pulmonary hypertension mouse models, confirming that elevated serum HIF-1α levels are positively correlated with mPAP^22^. Similarly, VEGF, which is implicated in vascular remodeling and pulmonary hypertension, exhibited elevated serum levels in CTD-PAH patients and correlated positively with mPAP.

Previous studies reported^23^ that VEGF expression is elevated in the serum of patients with hypoxia-associated PAH and correlates positively with PAP, which is consistent with our study’s findings. In our investigation, the serum VEGF concentration in the CTD-PAH group surpassed that of the CTD-non-PAH group and the HC, exhibiting a positive correlation with the mPAP (*r* = 0.88). The mechanism of VEGF action in CTD-PAH may mirror that observed in experimental animal models and other PAH types. Existing research suggests that VEGF stimulates the proliferation of vascular endothelial and smooth muscle cells by binding to specific receptors on pulmonary vascular endothelial cells. This process leads to the thickening of the walls of small pulmonary arteries, narrowing of the vascular lumen, and consequently, increased PVR, contributing to PAH development. The VEGF family and its receptors are distributed in various cell types, including alveolar epithelial cells, vascular endothelial cells, and airway smooth muscle cells. These proteins regulate crucial steps of angiogenesis, such as proliferation, survival, and migration of endothelial and precursor cells and promote angiogenesis^24^. Farkas et al.^25,26^ suggested that VEGF may play a dual role in pulmonary fibrosis by promoting fibrogenesis while also protecting vessels from excessive fibrosis-induced pressure. VEGF signaling pathways are closely intertwined with vascular remodeling activities^24,27^. The splicing variants of VEGF and its intricate signaling cascades are pivotal for maintaining pulmonary vascular homeostasis, and the VEGFR family plays a crucial role in severe PAH development. Analogous to animal models, the pathobiology of angioproliferative PAH in humans might also be influenced by VEGF subtypes and VEGFR-independent or VEGFR-dependent signaling pathways^26^. In such cases, VEGF not only participates in normal vasculogenesis but also may contribute to vascular remodeling and pulmonary hypertension development under pathological conditions by fostering abnormal proliferation and migration of vascular endothelial and smooth muscle cells. Our study’s results align with these findings, showing higher serum VEGF levels in CTD-PAH patients than in patients in the other groups, and these high levels were positively correlated with the mPAP measured by RHC as well as with the mRAP and PVR. These findings further underscore the potential role of VEGF in driving pathological processes in vascular smooth muscle and endothelial cells in CTD-PAH patients, culminating in pulmonary vascular remodeling and heightened PVR.

BNP is a hormone released in response to increased cardiac load and serves as a biomarker for heart failure, whereas the 6MWD is a commonly utilized clinical indicator for assessing patient exercise tolerance and cardiopulmonary function and is crucial in evaluating PAH. We analyzed the diagnostic efficacy of BNP using ROC curves, and our results suggest that HIF-1α and VEGF demonstrate more significant diagnostic capabilities and advantages. A supplementary Figure has been uploaded as an attached file. Our findings preliminarily affirm that the concentrations of HIF-1α and VEGF correlate with the clinical indicators BNP and the 6MWD in CTD-PAH patients, with both exhibiting a positive correlation with BNP and a negative correlation with the 6MWD. Moreover, the combination of these two biomarkers showed better diagnostic performance than BNP. This implies that CTD-PAH patients with elevated levels of HIF-1α and VEGF may experience a greater cardiac load and diminished cardiopulmonary function. The correlations between HIF-1α and VEGF and these clinical indicators suggest their potential utility as biomarkers for monitoring the condition and assessing the prognosis of CTD-PAH patients. Nonetheless, further research is imperative to elucidate the relationships between HIF-1α and VEGF levels and the prognosis of CTD-PAH patients. We hypothesize that their concentrations could be linked to the severity of vascular damage in CTD-PAH patients and that VEGF potentially influences the prognosis of these patients. In the future, a more comprehensive evaluation of disease progression and prognosis could be achieved by monitoring the association between these two biomarkers and patient survival rates, as well as observing patient responses under various treatment strategies.

A study demonstrated that the plasma concentration of HIF-1α was significantly elevated in SSc patients. The authors also utilized capillary microscopy to identify vascular injuries and highlighted the potential clinical application of HIF-1α in assessing microcirculation changes and vascular abnormalities in SSc patients. However, no relevant explanation was provided regarding PAH^28^. Our findings indicate that patients with CTD-PAH exhibit elevated serum levels of HIF-1α and VEGF. Moreover, HIF-1α and VEGF play significant roles in the elevation of PAP. The combination of these two biomarkers is a promising predictor of PAP in CTD-PAH patients and has the potential for early diagnosis, treatment guidance, and other aspects of CTD-PAH management.

The pathogenesis of CTD-PAH is notably intricate, with ample clinical research substantiating the significant roles played by a disordered immune system, immune cells, and cytokines in its onset and progression. Despite increasing clinician awareness of CTD-PAH, early diagnosis and prompt intervention remain critical in clinical practice. However, effective biomarkers are still lacking. Our study revealed significant interactions between HIF-1α, VEGF, and CTD-PAH, suggesting the involvement of these proteins in elevating PAP and potentially participating in pathological processes such as hypoxia, inflammation, and vascular remodeling. Analyzing the serum concentrations of VEGF and HIF-1α in CTD-PAH patients and their correlations with relevant clinical indicators offers new avenues for early diagnosis, targeted and personalized treatment, and risk assessment of CTD-PAH. Furthermore, this analysis provides a foundation for precision treatment, prognosis evaluation of PAH, identification of drug targets, and understanding of medicine responses.

However, the pathogenesis of CTD-PAH is multifaceted, and single biomarkers lack specificity. Our single-center retrospective cohort study was limited by the absence of large-scale, multicenter data to further validate and compare serum VEGF and HIF-1α levels in patients with other forms of pulmonary hypertension. Currently, no evidence suggests that the elevation of HIF-1α or VEGF is specific to CTD-PAH, nor that significant differences exist in their levels across various rheumatic immune diseases. Therefore, further research through more comprehensive studies is warranted to elucidate the pathological mechanisms and explore potential therapeutic approaches involving HIF-1α and VEGF in CTD-PAH patients.

## Electronic supplementary material

Below is the link to the electronic supplementary material.

Supplementary Material 1

## Data Availability

The datasets used and/or analyzed during the current study are available from corresponding author on reasonable request.

## Abbreviations

PAH: Pulmonary arterial hypertension
CTD: Connective tissue disease
CTD-PAH: Connective tissue disease-associated pulmonary arterial hypertension
CTD-non-PAH: Connective tissue disease patients without pulmonary arterial hypertension
HIF-1α: Hypoxia-inducible factor-1α
VEGF: Vascular endothelial growth factor
RHC: Right heart catheterization
HC: Healthy control group
ELISA: Enzyme linked immunosorbent assay
SLE: Systemic lupus erythematosus
SSc: Systemic sclerosis
RA: Rheumatoid arthritis
ROC: Receiver operating characteristic
SS: Sjögren’s syndrome
MCTD: Mixed connective tissue disease
mPAP: Mean pulmonary arterial pressure
PAP: Pulmonary arterial pressure
NT-proBNP: N-terminal probrain natriuretic peptide
6MWD: 6-Minute Walk Distance
BNP: B-type natriuretic peptide
NT-proBNP: N-terminal pro-b-type natriuretic peptide
PAWP: Pulmonary arterial wedge pressure
PVR: Pulmonary vascular resistance
RAP: Right atrial pressure
CI: Cardiac index
SD: Standard deviation
IQR: Interquartile range
AUC: Area under the curve
mRAP: Mean right atrial pressure
