# Supplementary figures and images for: Elevated serum levels of HIF-1α and VEGF as potential biomarkers in connective tissue disease-associated pulmonary arterial hypertension
Source: Sci Rep. 2025 Feb 13;15:5410. doi: 10.1038/s41598-025-89130-w (PMC11825724; doi:10.1038/s41598-025-89130-w)

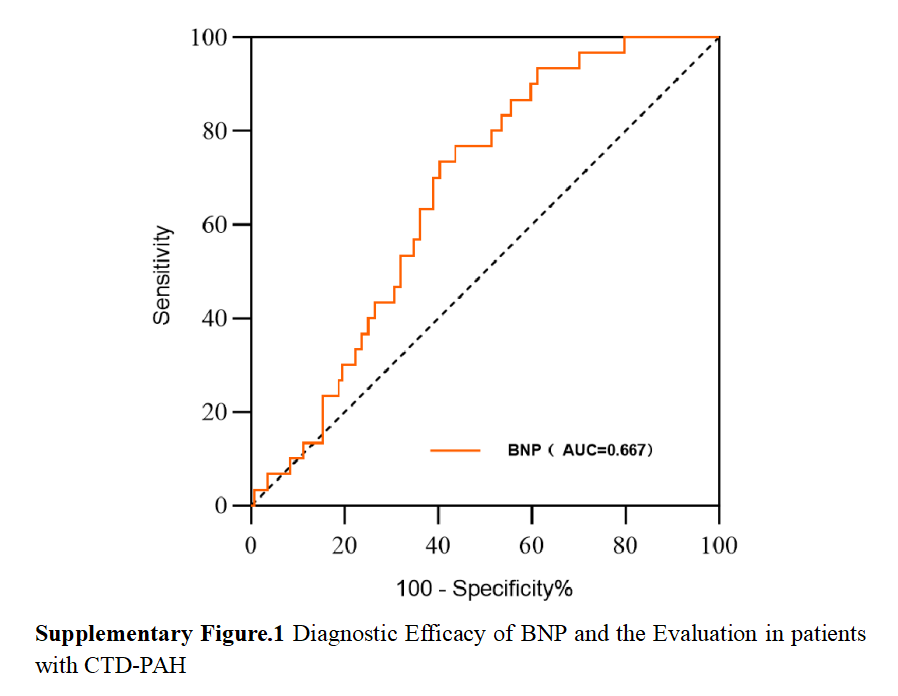

Supplement: Supplementary file 1 — Supplementary Material 1 [file 41598_2025_89130_MOESM1_ESM.png]
